# Innovative perspective for the cleaning of historical iron heritage: novel bio-organogel for the combined removal of undesired organic coatings and corrosion

**DOI:** 10.1186/s40494-024-01288-0

**Published:** 2024-06-05

**Authors:** Arianna Passaretti, Luana Cuvillier, Giorgia Sciutto, Edith Joseph

**Affiliations:** 1 Haute Ecole Arc Conservation-Restauration, HES-SO University of Applied Sciences and Arts Western Switzerland, Espace de l’Europe 11, 2000 Neuchâtel, Switzerland; 2 Laboratory of Technologies for Heritage Materials, Institute of Chemistry, University of Neuchâtel, Av. Bellevaux 51, 2000 Neuchâtel, Switzerland; 3 Department of Chemistry, University of Bologna, Ravenna Campus, Via Guaccimanni, 48121 Ravenna, Italy

**Keywords:** Green cleaning, Organogels, Bio-solvents, Deferoxamine B, Organic coatings, Iron corrosion, Metal heritage

## Abstract

An innovative green organogel was designed to simultaneously tackle inorganic compounds (i.e., iron corrosion) and organic substances (i.e., acrylic coatings) as undesired materials possibly present on the surface of altered indoor metal artworks. Poly-3-hydroxybutyrate (PHB), ethyl lactate (EL), and deferoxamine B (DFO) were employed in the formulation as thickening agent, organic solvent, and complexing agent, respectively, aiming to propose a sustainable and less harmful chemical cleaning method for metal care. The components were selected because they are bio-sourced, renewable, biodegradable, and non- or low-toxic materials. A multi-modal protocol of analysis was carried out to characterise the newly designed PHB-EL-DFO organogel. The cleaning performance of the novel formulation was assessed on mild steel mock-ups presenting both corrosion and organic coating to be removed. The conducted multi-analytical approach verified that the PHB-EL-DFO gel was able to tackle the two undesired materials simultaneously in an adjustable and easy-to-use way thanks to a modular application.

**Supplementary Information:**

The online version contains supplementary material available at 10.1186/s40494-024-01288-0.

## Introduction

Ferrous alloys naturally tend towards the spontaneous phenomenon of corrosion [1, 2]. In indoor environments, the process can be influenced by factors such as artificial and natural light, dust, and several volatile compounds present in the surrounding atmosphere (e.g., H_2_S, COS), yet it is primarily driven by ambient temperature and relative humidity fluctuations [3]. Indeed, the phenomenon is provoked on indoor historical ferrous heritage in the presence of an electrolyte (i.e., condensed water) and an oxidant (i.e., oxygen in the air) and follows the so-called “wet-dry” cycle [3, 4]. The whole cyclic process of atmospheric corrosion affecting iron can be expressed by the chemical Eq. 4Fe + 3O_2_ + 2H_2_O → 4FeOOH [1]. Various iron oxyhydroxides and oxides can be formed through the phenomenon, typically, goethite (α-FeOOH), akageneite (β-FeOOH), lepidocrocite (γ-FeOOH), feroxyhyte (δ-FeOOH), ferrihydrite (hydrated oxyhydroxide of iron), maghemite (γ-Fe_2_O_3_), and magnetite (Fe_3_O_4_) [5, 6]. The possible presence of reactive species, such as ferrihydrite, akageneite, or lepidocrocite, can cause degradation and irreversible damages to the substrate due to the formation of localised pitting corrosion or uniform alteration of the metallic surface [7–9].

Therefore, a preventive method against corrosion consists of limiting the interaction between the metal surface and the surrounding environment through the application of organic coatings [10]. In the case of indoor historical iron-based heritage, mainly microcrystalline waxes and acrylic resins are used by CRs for its protection [11]. However, these materials are equally sensitive to detrimental factors over time, resulting in negative consequences mainly related to aesthetic issues (i.e., yellowing phenomena) and protection failure (i.e., cracking, detachment) that leads to the exposure of the underlying metal to corrosive agents [12, 13].

Generally, the preservation of the original surface of metal artefacts is prioritised due to their artistic, historical, aesthetic, and monetary value despite the possible presence of alterations [14]. Nevertheless, when the appearance, functionality, or long-term conservation are endangered by artwork surface conditions, it is inevitable to evaluate the alterations exhibited (e.g., noble or detrimental patina, protective properties of organic coatings) and consider the removal of compounds no longer acceptable for aestheticism and preservation purposes, including corrosion and/or aged filming products [9, 14–17]. When such interventions are required, compromised organic coatings are frequently found associated with altered underlying metal substrates over time [15, 16, 18, 19]. Usually, a preliminary removal of aged organic materials is mandatory to tackle the reactive corrosion present within and underneath; hence two—or more—separately addressed cleaning approaches are necessary. Thus, a method targeting simultaneously altered protective films and corrosion products could be of interest to achieve a versatile cleaning action.

In this paper, an innovative multi-functional bio-organogel is proposed to tackle concurrently iron corrosion products and undesired organic coatings possibly present on indoor historical metal artefacts. The first gel formulations were introduced by Richard Wolbers to the world of art conservation for the high-controlled and less invasive treatment of paintings [20, 21], then becoming indispensable cleaning methods in several other fields, including stone, paper, and textile care [22–25]. Specifically, the retention in the gel network provides a more controlled release of the active solution and ease in handling during application and clean-up [26]. Furthermore, highly localised cleaning and adjustability can be addressed according to the needs of the specific case. Finally, the confinement of the solution in a gelled matrix induces a reduction of active agents’ quantities (e.g., hampering solvent evaporation), resulting additionally in a higher safety for the operator due to a minor exposure to potentially harmful active agents (e.g., solvent vapours) [27]. Nevertheless, the use of gels on metals is still anecdotal in spite of the need for spatially precise treatments, when working on composite objects or gilded artworks, or for the controlled release of active solutions and solvents on highly sensitive corroded metals [28–31]. Furthermore, chemicals employed during conservation interventions (e.g., solvents, acids, chelators) can be corrosive for metals if not accurately handled and retained [32, 33]. However, encouraging research demonstrated the reliability and suitability of gels as cleaning systems for the adjustable and controlled removal of altered organic films or harmful corrosion [29, 30, 34, 35].

The innovative formulation, presented in this study, was designed with the aim of simultaneously removing undesired organic substances and inorganic compounds from corroded steel substrates. Specifically, the removal of an acrylic varnish (i.e., Paraloid® B72) and the underlying corrosion, generally composed of iron oxyhydroxides (i.e., lepidocrocite, goethite), was studied as representative for protective coatings and alterations often present on indoor historical iron alloys. These targets were selected in accordance with the replies obtained through a survey addressed to metal conservator-restorers (CRs) and spread worldwide by authors in 2020 [36].

The field of heritage conservation makes no exception in the global rise of attention towards greener attitudes, as confirmed by the increasing number of harmless and more sustainable solutions and practices sought in the last decades [26, 37]. In this scenario, exclusively biologically derived and biodegradable components were employed in the gel formulation. In particular, the system was designed employing the bio-polymer poly-3-hydroxybutyrate (PHB) because produced from renewable sources, non-toxic, and readily biodegradable into carbon dioxide, water, and biomass [26]. PHB has already been exploited as a formulation carrier in art conservation and tested to be easily recoverable after the use, enhancing the greenness of this polymer [38, 39]. Poly-3-hydroxybutyrate can be dissolved by the bio-solvent ethyl lactate (EL), as verified by previous research [40]. This organic solvent was selected in this research due to its demonstrated sustainability, being bio-sourced and biodegradable, and, despite the required use of PPE for handling, lower toxicity (e.g., inhalation threshold) and hazard (e.g., lower flammability and volatility) towards operators compared to other conventional solvents such as methyl ethyl ketone (MEK) and acetone [41–43]. Furthermore, ethyl lactate is a polar protic solvent able to dissolve acrylic resins, such as Paraloid® B72, which is the organic coating targeted for this study [44]. In parallel, the natural siderophore deferoxamine B (DFO) was exploited as a green alternative to traditional hazardous and pollutant complexing agents employed extensively in metal care, such as ethylenediaminetetraacetic acid (EDTA). The siderophore is a hexadentate ligand naturally produced by bacteria, non-toxic, and fully biodegradable [45, 46]. DFO presents three bidentate hydroxamate functional groups (–CONHO–), which are the binding sites for the coordination with metal ions [46, 47]. Its high affinity for ferric ion Fe(III) with at a molar ratio of 1:1, as expressed by the stability constant logβ = 30.4 [48], propose DFO as a promising alternative green ligand for the removal of iron corrosion products, as already tested in art conservation [49, 50].

## Experimental

### Reagents

For the synthesis of the gel formulation, poly-3-hydroxybutyrate (PHB, CAS number: 29435-48-1) was purchased from Biomer, ethyl lactate (CAS number: 97-64-3) from Sigma Aldrich, and deferoxamine mesylate salt (Desferal®) (DFO, CAS number: 138-14-7) from Novartis.

For the preparation of model samples, mild steel coupons (50 × 50 × 2 mm^3^) from Tartaix Métaux Outillage, iron (III) chloride hexahydrate from Carlo Erba (CAS number: 10025-77-1), Paraloid® B72 from CTS, ethanol (96.0–97.2%, CAS number: 64-17-5), and ethyl acetate (≥ 99.7%, CAS number: 141-78-6) from Sigma Aldrich were employed.

### Mock-up preparation

Mild steel coupons were initially degreased with ethanol and acetone and then chemically aged adjusting the protocol ASTM G48-11(2015) (Ferric Chloride Pitting Test). The metal pieces were immersed in a 11% w/v solution of iron (III) chloride hexahydrate for 24 h, then rinsed with deionised water, and left to dry in uncontrolled conditions. The protocol allowed to obtain a layer of orange-brown coloured corrosion, homogeneously distributed on the surface of steel sheets. Finally, the coupons were coated by brush with two thin criss-cross layers of Paraloid® B72 (10% w/v solution in ethyl acetate) and left to dry. Although the samples were not prepared through the natural aging of coated metal pieces, the application of Paraloid® B72 on corroded, thus porous, substrates allowed to obtain model samples with the coating not only present on the surface but also within the corrosion itself, enhancing the validity of the mock-ups used.

The mock-ups were characterised by Raman spectroscopy to verify the nature of the corrosion phases obtained through chemical ageing. The analysis was possible as Paraloid® B72 is a transparent material and permits the interaction of the excitation laser with the metal surface.

### PHB-EL-DFO organogel formulation

**Fig. 1:**
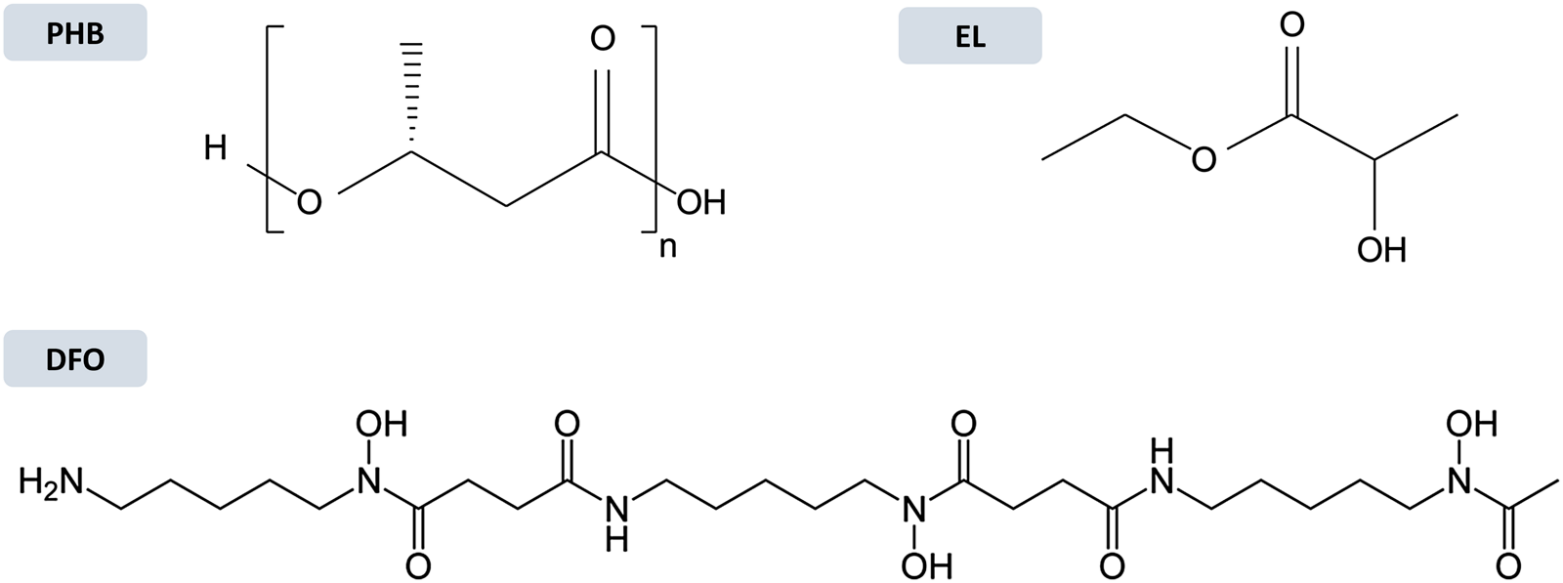
Structural formula of PHB-EL-DFO gel components: poly-3-hydroxybutyrate (PHB), ethyl lactate (EL), and deferoxamine B (DFO)

The PHB-EL-DFO gel formulation was prepared using a 20% w/v solution of deferoxamine (DFO) in deionised water and ethyl lactate (EL) in a volume ratio of 1:6. The polymer poly-3-hydroxybutyrate (PHB) was then added at 7% w/v concentration. The final concentration of the DFO solution was 4 × 10^–2^ M, which is a value in the range of other exploitations of this ligand in art conservation [24, 51, 52]. The mixture was stirred in a glass petri dish at about 110 °C until the gel was formed and let cool down to room temperature before application (Fig. 1).

The complexing action of deferoxamine for Fe(III) is functional at the pH measured in the PHB-EL-DFO gel (i.e. pH 4.8), and it is not altered by the temperature reached during the preparation of the gel (i.e., 100–110 °C) [46, 52, 53]. In addition, the commercial product used (Desferal®, Novartis) is in the form of mesylate salt that needs to be solubilised to allow the complexation. Solubility tests on Desferal® were carried out with ethyl lactate, ethanol, and deionised water; only the latter yielded an active DFO solution. Therefore, the minimum amount of water required for DFO solubilisation was estimated and the gel system amended accordingly.

### Cleaning protocol

Before evaluating the cleaning performance of the PHB-EL-DFO gel on mock-ups, a preliminary test was performed without DFO amendment. A PHB-EL gel prepared following the same protocol was applied three times for 30 min each on a bare mild steel coupon to check if any negative impact could occur on the metal substrate, mainly due to the organic solvent present (i.e., ethyl lactate). Despite the slightly acidic pH (i.e., 4.8) of the formulation, similar to that of PHB-EL-DFO gel, no alteration was evidenced on the mock-up surface comparing optical microscopy images collected before and after application of the PHB-EL gel. Furthermore, the comparison of CIELab coordinates recorded for the bare mild steel coupon before and after gel application showed a negligible colour difference calculated as ΔE* = 0.16 (± 0.42), confirming the safety of the formulation on the metal substrate. This finding is in line with the previous literature reporting the use of the organic solvent on metals [54–56].

The cleaning performance of the PHB-EL-DFO gel was investigated with three different application times. The organogel was assayed on triplicate mock-ups using intervals of 10, 20, and 30 min and by two consecutive applications, renewing the gel for each application. After cutting in a 5-cm-diameter circle-quarter shape with a metallic spatula, the gel was applied onto mock-ups and gently pressed with the same tool to maximise the contact and avoid air bubbles between the formulation and the metal surface. On each mock-up, four equal areas were defined: three were cleaned at intervals of 10, 20, and 30 min respectively, and the remaining area was left untreated. After application, the gels were removed in one stroke by a spatula. Eventually, the mock-up surface was cleared with ethanol 70% v/v using a cotton swab to remove possible gel residues, swollen Paraloid® B72, and iron-DFO complexes formed during the application.

### Equipment

The mechanical features of the PHB-EL-DFO formulation were investigated by amplitude sweep tests with an Anton Paar rheometer MCR 102. The analysis configuration included a parallel-plate (25 mm) measuring system, temperature at 25 °C, pre-set force for the detection of the gel sample surface at 1N, angular frequency (ω) of 5 s^−1^, 25 data points, and shear strain amplitude (γ) range of 0.001—100%. A PHB-EL-DFO gel and a re-heated one (i.e., 100–110 °C) were examined to evaluate thermoreversibility.

A ZEISS Axioscop 2 MAT optical microscope equipped with an Axiocam 305 color camera was used to observe and monitor the surface of mock-ups beforehand and during the cleaning protocol. EDF (Extended Depth of Focus) images were collected by manual focus drive using the ZEISS software ZEN core 3.2. The examination was conducted under reflected light in brightfield mode and under UV light with a fluorescence filter at 365–420 nm, employing 20× and 50× magnification objectives (ZEISS Epiplan). The parameters for exposure time and white balance were set automatically by the software.

Cryo-Scanning Electron Microscopy (Cryo-SEM) was performed with a Quanta FEG-250 SEM (Thermo Fisher Scientific Inc.) instrument coupled with a cryo-chamber Quorum PP3010 Cryo-Unit. The technique allowed to observe at high resolution the inner structure of the PHB-EL-DFO gel. Still-moist gels were placed on an aluminium stub, frozen by liquid nitrogen slush, and transferred into the cryo-vacuum chamber precooled to − 140 °C. The gels were then fractured and sublimated for 40 min at − 60 °C to remove ice contamination. Finally, the samples were coated by sputtering with platinum (15 s coating at 10 mA current) in an argon atmosphere at − 140 °C. SEM secondary electrons (SE) images of gel surfaces and fractures were captured with a voltage of 6 kV and a working distance of about 13 mm.

Thermogravimetric analysis (TGA) was performed to investigate the capacity of the designed systems to retain the loaded solvents and active solutions by comparing the evaporation rates obtained for neat liquids and gelled formulations. A Perkin–Elmer TGA 7 thermogravimeter was employed and Pyris™ software (Perkin–Elmer) was used for data acquisition. About 20 mg of samples were used and placed in open aluminium pans. The analysis consisted of an isothermal run at 40 °C for 60 min performed under nitrogen flow. The temperature of 40 °C was reached by a programming heating rate of 40 °C/min starting from the default system temperature of 25 °C. The experimental temperature of 40 °C was selected because it is the lowest condition within the operational limits of the TGA instrument employed and is in accordance with the previous literature as the most representative for CRs working conditions [34, 38].

A Konica-Minolta CM26d portable spectrophotometer was used to collect colourimetric data on the mock-ups before and after each gel application to evaluate potential chemical alterations caused by the gel in a preliminary phase and to ascertain the impact of application time on cleaning efficiency. Collected data are correlated to colorimetric measurements acquired on non-corroded and non-coated mild steel coupons as controls. The colourimetric data were recorded using the CIELab colour space with standard illuminant D65, d/8° geometry, 10° standard observer, and instrument window size of 3 mm. Three different spots were examined within each treated area, with triplicate samples taken, resulting in a total of nine measurements for each cleaning treatment. Collected Specular Component Excluded (SCE) data were averaged and elaborated. Colour changes are reported and discussed as L, a, b coordinates and ΔE* value, which was calculated, according to EN 15886:2010, as $\Delta {E}^{*}= \sqrt{{\left({L}_{2}^{*}-{L}_{1}^{*}\right)}^{2}+{\left({a}_{2}^{*}-{a}_{1}^{*}\right)}^{2}+{\left({b}_{2}^{*}-{b}_{1}^{*}\right)}^{2}}$, where ${L}_{1}^{*}, {a}_{1}^{*},{b}_{1}^{*}$ are the CIELab values of the first measurement, and ${L}_{2}^{*}, {a}_{2}^{*},{b}_{2}^{*}$ those of the second one. Colorimetric data were further processed by Tukey’s HSD test to evaluate the statistical significance among the several application times explored.

X-ray fluorescence (XRF) analysis was carried out by means of a Bruker ARTAX portable XRF spectrometer. The device is provided with a low-power metal-ceramic-type X-ray tube with a Mo anode and a Peltier-cooled silicon drift detector (SDD). The X-ray beam was focused on the gel samples before and after application on mock-ups using a CCD camera and a motor-driven XYZ stage. The analysis was set with a potential at 30 kV, current intensity at 700 μA, a life-time of 50 s, and a spot of analysis lower than 1 mm^2^.

A Renishaw Virsa™ Raman Analyser was employed for single-point analysis with 3–4 cm^−1^ spectral resolution, using a 785 nm excitation diode laser and a 50× Nikon objective. Still-moist gel samples were characterised before and after application on mock-ups in the spectral range 500–1600 cm^−1^, setting the analysis at 30 mW, 10 accumulations of 1 s. The metal mock-ups were examined after chemical ageing, after Paraloid® B72 coating, and at the end of the cleaning protocol. Related data were collected in the spectral ranges 50–1250 cm^−1^ and 500–1600 cm^−1^ at 3 mW, 100 accumulations of 3 s. Reference spectra of iron (III) chloride hexahydrate and Paraloid® B72 were collected with the same set-up of analysis. Data elaboration was performed on Thermo Grams/AI 8.0™ suite software (Thermo Fischer Scientific). Savitzky Golay derivative (third-order polynomial and 11 datapoint gaps) was applied to spectra to ease their readability.

Micro-Fourier-transform infrared spectroscopy (FTIR) was performed with a Thermo Nicolet™ iN10 MX infrared imaging microscope, equipped with a mercury-cadmium-telluride (MCT) detector cooled by liquid nitrogen. Mock-ups were analysed by single-point analysis in reflectance mode in the spectral range 4000–675 cm^−1^, with an optical aperture of 400 × 400 μm, a spectral resolution of 4 cm^−1^ and 64 scans. Additional mapping analysis was run with the same parameters but lowering the number of scans up to 16. Three maps were acquired per each treatment triplicate employing a set-up of 200 × 200 μm step-size and 10 × 10 steps. Data were collected with the dedicated software OMNIC Picta™ and elaborated with OMNIC™ (Thermo Fisher Scientific). Principal Component Analysis (PCA) was performed using nine single-point FTIR spectra recorded on bare mild steel coupons and three spectra per replicate (for a total of nine spectra per used gel) to evaluate the cleaning performance according to the application time. For multivariate analysis, the original acquisition range was limited to the fingerprint region of 2000–1000 cm^−1^, which was of interest in this research study. The resulting spectra were then pre-treated by standard normal variate (SNV) transform and by column centring before performing PCA by CAT (Chemometric Agile Tool) software [57].

## Results and discussion

### Characterisation of the PHB-EL-DFO gel

**Fig. 2:**
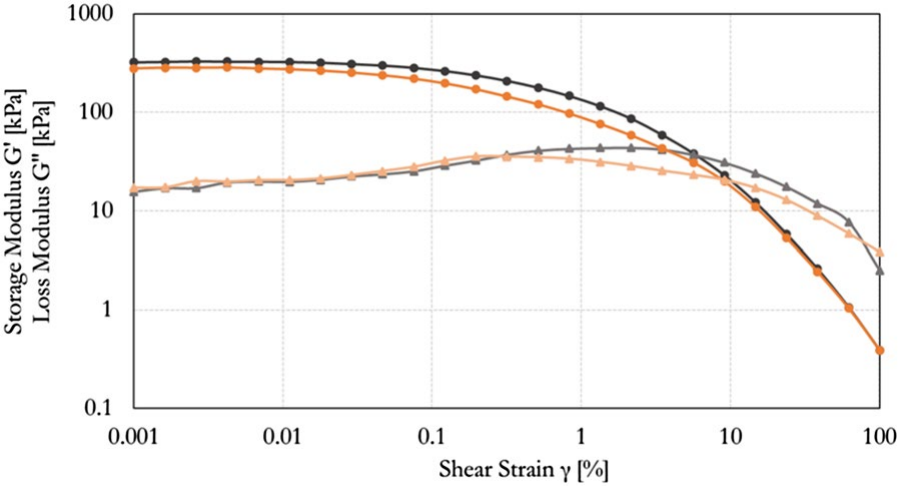
S Strain dependence of storage modulus Gʹ (circles, darker hue) and loss modulus G″ (triangles, lighter hue). The amplitude sweep curves for PHB-EL-DFO formulation and re-heated formulation are plotted in black and orange, respectively

The mechanical properties of the PHB-EL-DFO formulation were investigated via rheology using amplitude sweep analysis. The instrument recorded values for the storage modulus Gʹ (i.e., formulation stiffness) approximately an order of magnitude higher than the loss modulus G″ (i.e., formulation viscosity) over the linear viscoelastic (LVE) region (Fig. 2, black plot). This region is illustrated as a plateau trend for the storage G' modulus curve and represents the range of shear strain tested for which the sample structure is not destroyed. The result obtained in the LVE region with Gʹ > G″ is distinctive for viscoelastic solids, characterised by strong interaction forces, and describes the PHB-EL-DFO samples as gel-like structured in rheology [58]. After leaving the LVE region, a gradual drop of the curve occurs, indicating a non-brittle fracturing behaviour of the gel until reaching the crossover point (i.e., Gʹ = G″). Considering the intended use of the PHB-EL-DFO formulation on metal artefacts, the registered stiffness and response to shear strain indicate appropriate characteristics such as easy handling for operators and potentially no residues after gel removal. The formulation stiffness might also imply poor adaptability to the probable unevenness of metal surfaces (e.g., inlays, corrosion roughness). Nonetheless, the rheological behaviour registered for the PHB-EL-DFO gel was in line with previous literature related to similar PHB-based formulations that have been successfully applied in art conservation, yet not amended with the DFO siderophore [35, 38].

Finally, a PHB-EL-DFO gel (Fig. 2, black plot) and a re-heated one (Fig. 2, orange plot) were examined by amplitude sweep to evaluate the thermoreversibility of the system. The comparison between the resulting plots did not highlight a significant difference in the response when subjected to shear strain. As displayed in Fig. 2, the tested samples had comparable curve trends, similar values of Gʹ and G″ moduli in the LVE region and crossover points (i.e., Gʹ = G″). The observed unaltered behaviour verified the thermoreversibility of the PHB-EL-DFO system.

**Fig. 3:**
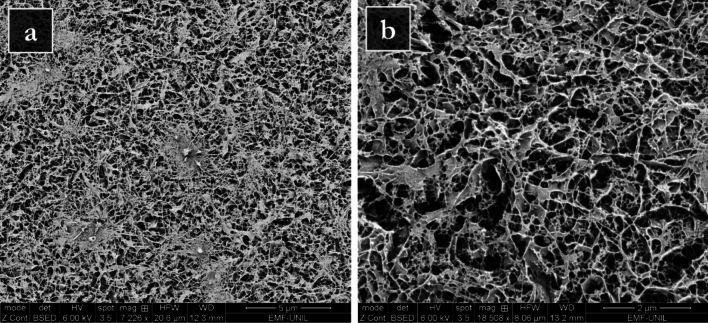
Cryo-SEM back-scattered electrons (BSE) images of PHB-EL-DFO inner structure after sublimation etching and platinum coating. The scale bar indicates 5 µm and 2 µm in image a and b, respectively

Cryo-scanning electron microscopy (cryo-SEM) analysis allowed the examination of the inner structure of still-moist PHB-EL-DFO gels and provided an explanation for the observed rheological behaviour of the system. This analysis was preferred over conventional SEM since the cryo-technique enables the instant freezing of samples by liquid nitrogen slush to effectively remove the liquid fraction enclosed in gels without modifying the samples’ structure [59]. The resulting Cryo-SEM images, acquired in back-scattered electrons (BSE) mode on the gels, are reported in Fig. 3. In general, the structure of the PHB-EL-DFO formulation was characterised by a PHB-based matrix densely interconnected, as visible in Fig. 3a. The gels presented a non-regular plate-like structure interspersed by blocky pores. The cavities displayed a diagonal aperture that could be measured in the order of 10^–1^–10^–2^ µm (Fig. 3b). However, pores were not regularly dispersed in the PHB-EL-DFO samples (Fig. 3a). Indeed, the cryo-SEM images displayed denser matrix areas, where cavities were barely visible at the magnification employed for the analysis. Such compact zones and the overall small size of pores in the PHB-EL-DFO gel would corroborate rheological measurements, indicating a high stiffness for the analysed samples.

**Fig. 4:**
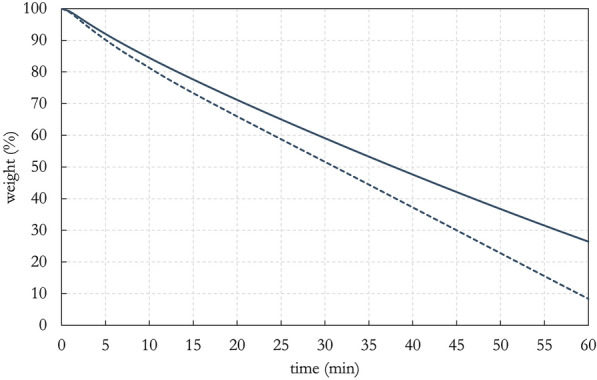
Isothermal TGA scan at 40 °C in nitrogen atmosphere comparing the weight loss of free EL-DFO-water solution (dashed line) to the PHB-EL-DFO gel (solid line). Data were processed considering samples’ liquid fraction solely

Finally, the PHB-EL-DFO gel was studied by thermogravimetric analysis (TGA) to evaluate the evaporation of the liquid phase englobed compared to the unrestrained DFO solution (i.e., 20% in deionised water) in ethyl lactate (i.e., 1:6 v/v). As the analysis was an isothermal run at 40 °C, the recorded weight loss could be attributed entirely to the evaporation of the liquid phase present in the samples, and not to poly-3-hydroxybutyrate or deferoxamine powders, which are in a solid state at the set temperature [60, 61]. Consequently, results shown in Fig. 4 were calculated as weight loss percentages considering exclusively the samples’ liquid fraction to correctly interpret the obtained data. The analysis verified that the liquid portion in the free EL-DFO-water solution (Fig. 4, dashed line) was more prone to evaporation compared to when restrained in the PHB system (Fig. 4, solid line) over time in the set conditions. In particular, after 60 min of isothermal run at 40 °C, the weight of the unstrained solution dropped down to 9% of its initial value. In comparison, the solution residually retained in the gelled system was three times more, reaching approximately 28% of its starting weight. The PHB-EL-DFO formulation demonstrated an ability to hinder the unwanted loss of active solution in the atmosphere in the explored conditions, possibly reducing the quantities of liquid components required during a similar cleaning intervention.

### Evaluation of the cleaning action

The performance of the PHB-EL-DFO gel was assessed on mock-ups prepared as corroded mild steel coupons coated with Paraloid® B72, following the cleaning protocol described in “Cleaning protocol” section. This procedure was selected because it ensured that only application time was the variable parameter, keeping constant factors such as the quantity of the complexing agent provided at each cleaning step, treated mock-up area dimensions, and post-cleaning cotton-swabbing.

**Fig. 5:**
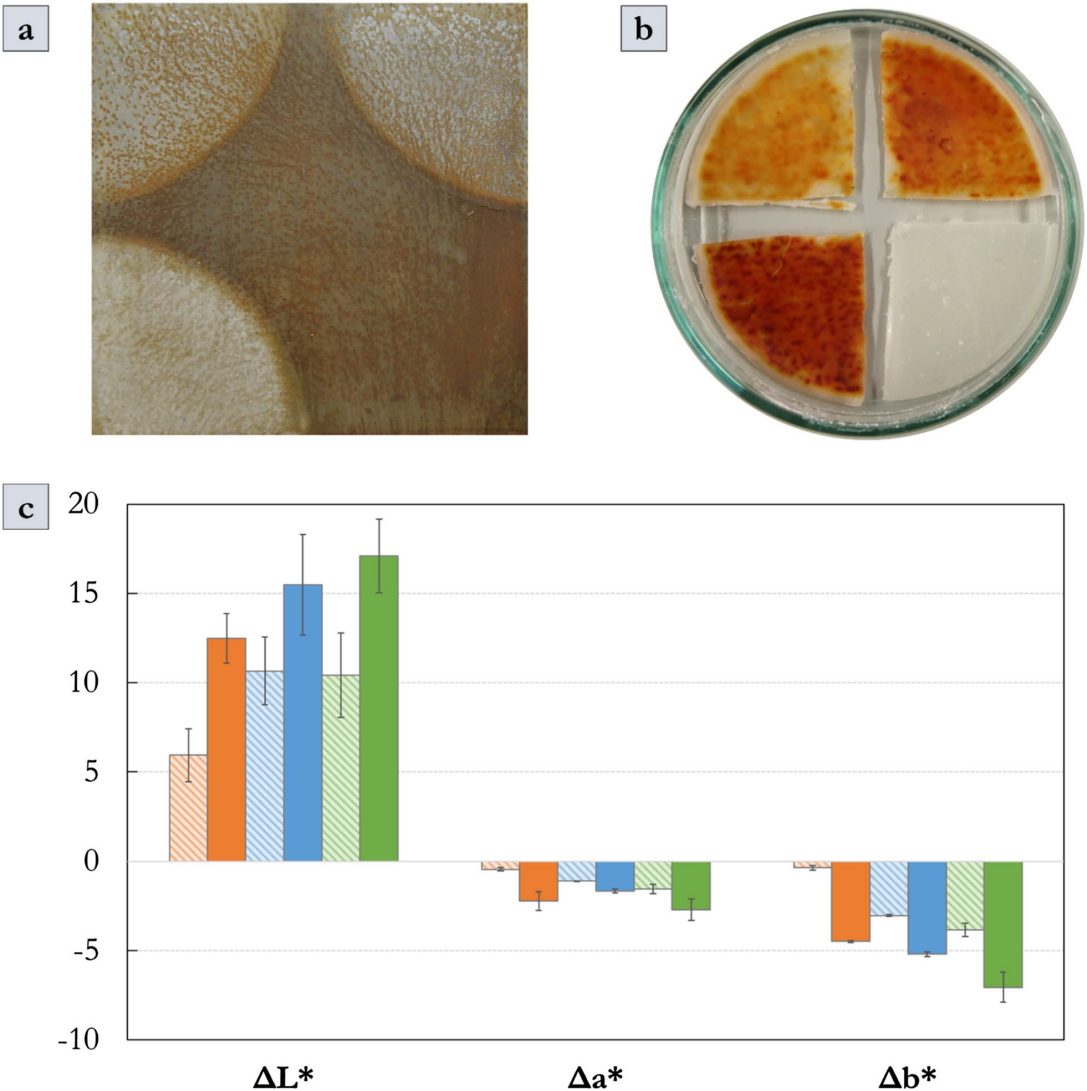
**a** Chemically aged mild steel mock-up, coated with Paraloid® B72, partially cleaned by PHB-EL-DFO gel. Cleaning outcomes after two gel applications of 10 (top left corner), 20 (top right corner), and 30 (bottom left corner) minutes, respectively. Bottom-right sector left untreated as a control. **b** PHB-EL-DFO gels removed from the mock-up after 10 (top-left sector), 20 (top-right sector), and 30 (bottom-left sector) minutes, and PHB-EL-DFO gel not applied (bottom-right sector) as a control. **c** Variation of CIELab coordinate values of the mock-up sectors comparing before and after each cleaning treatment step. ΔL*, Δa* and Δb* values are reported, correlated with their standard deviation, in the bar chart. Data for the cleaning protocols of 10 (orange), 20 (blue) and 30 (green) minutes are reported as striped- or solid-coloured bars to display one or two gel applications, respectively

Throughout the cleaning process, the action of the formulation was visually evident and increased with the number of gel renewals for all different application times (Fig. 5a). Indeed, the formulation changed from whitish to bright red coloured indicating the possible formation of iron-DFO complexes, as reported in the literature [51] (Fig. 5b).

After the removal of gels and cotton swabbing of the treated zones, optical microscopy easily emphasised the mock-up surface features. In agreement with visual appearance, the 30-min treatment with gel renewal yielded a more homogeneous and deeper cleaning, showing less rusted but more metallic grey-coloured areas, resembling the original appearance of the steel mock-up. No clear traces of gel residues could be visually evidenced in all treated mock-up areas (SM-Figure 1). Finally, microscopic observation under UV light suggested the absence of evident coating remains after 20- and especially 30-min treatments (SM-Figure 1), yet Fourier-transform infrared spectroscopy (FTIR) was exploited in this study to better detect the presence of residual coating on the treated areas, as discussed in the next paragraphs.

Colorimetric data collected on the mock-up triplicates, before and after each gel application, are consistent with the interpretation achieved by optical microscopy. Comparing the CIELab coordinates, the treated surfaces gained brightness, as expressed by the positive ΔL* values. On the contrary, the negative Δa* and Δb* stated a loss in red and yellow shades, respectively, ascribable to the removal of iron corrosion compounds. Overall, this result was similar for all the different application timings and was consistent with the renewal of the gel (Fig. 5c). Relating cleaned mock-ups to non-corroded mild steel coupons, the colour difference was still perceptible with the naked eye since above the threshold of ΔE* ≈ 2.3 [62]. In general, ΔE* decreased with gel reiteration and for longer application times. Namely, ΔE* was calculated as 19.36 (± 3.02), 15.26 (± 3.49), and 13.47 (± 2.60) for the mock-up areas treated by two applications of 10, 20, and 30 min, respectively. The result showed that also when opting for long application times (i.e., 30 min in this study), the gelled system could provide a modulable cleaning through reiterations, avoiding unsought overcleaning and full recovery of the bare metallic appearance, which is not commonly sought in the case of historical metal objects [51, 63].

As a final evaluation, Tukey’s HSD test was applied to the colorimetric data to verify the relevance of the differences noticed among the several times and gel applications explored. The analysis demonstrated that both application time and renewal are statistically relevant for the discrimination of the cleaning outcomes when considering L* (i.e., brightness) and a* (i.e., red component) coordinates, but not for b* (i.e., yellow component). It is remarkable that, when comparing one application of 20 min to two applications of 10 min each (i.e., for a total contact time of 20 min), their difference was statistically relevant (p < 0.05), possibly showing that gel renewal was a more determinant factor rather than application time for the final cleaning outcome. This observation would be explained by both the renewal of the active agent (i.e., ethyl lactate and DFO), when applying another gel, and by the intermediate clearance step that could have removed residual iron-DFO complexes and swollen Paraloid® B72 when operating by the 10-min protocol. In line with such observation, the statistical test did not evidence clear discrimination between the outcomes achieved by two gel applications of 20 or 30 min for L* and a* coordinates (p > 0.05).

Elemental and molecular analyses were performed on the gel surface before and after application on the mock-ups to investigate the performance of the innovative cleaning system. In particular, here are presented the results obtained after one application of 10 min, considered indicative to appreciate the efficiency of the gelled systems already when employing a short-time protocol.

**Fig. 6:**
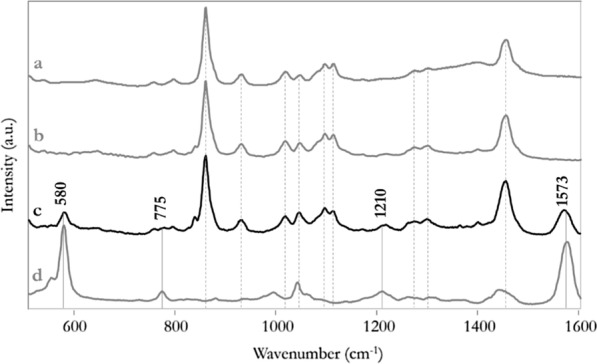
Raman spectra of ethyl lactate (**a**), PHB-EL-DFO still-moist gel before application (**b**), PHB-EL-DFO still-moist gel after one application of 10 min (**c**), and Fe-DFO complexes (**d**). Raman bands related to ethyl lactate (**a**) and Fe-DFO complexes (**d**) are highlighted by dashed and solid lines, respectively. The wavenumbers of Raman peaks diagnostic for Fe-DFO complexes are reported in the figure

X-ray fluorescence spectroscopy revealed the presence of iron on the gel only after the application on the mock-ups, thanks to the peaks detected at approximately 6.4 and 7.0 keV corresponding to the Kα1 and Kα2 lines characteristic for iron, respectively. Raman spectroscopy, applied to the used organogels, could attribute this outcome to the presence of iron-DFO complexes that were formed during the cleaning process and confined within the gel (Fig. 6). Indeed, the analysis, performed on still-moist gels after a 10-min application on the mock-ups, revealed characteristic Raman bands distinctive for Fe-DFO complexes around 580 and 1573 cm^−1^, respectively linked to Fe–O stretching and C=N stretching (i.e., amide functional group) vibrations (Fig. 6c), in accordance with the reference spectrum obtained in this work (Fig. 4d) and the previous literature [64].

**Fig. 7:**
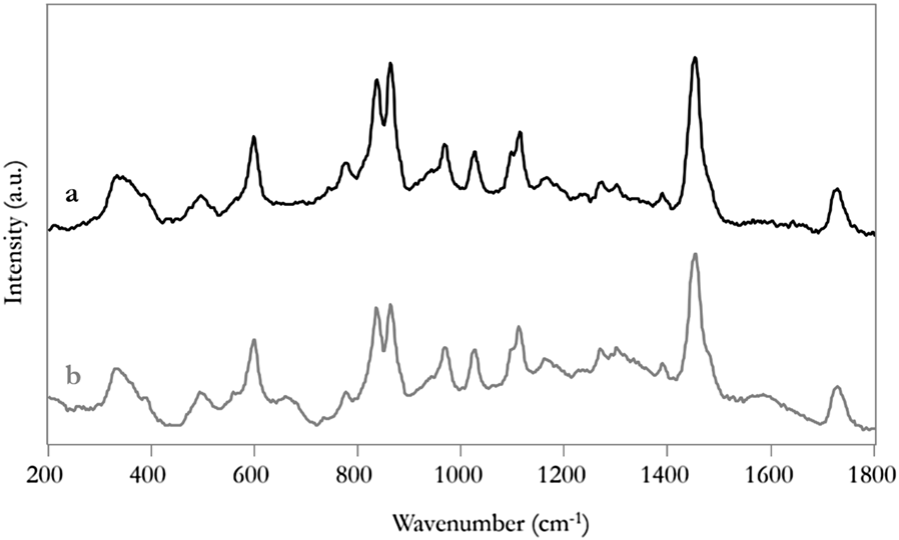
Raman spectra of Paraloid® B72 (**a**) and extraction residue from PHB-EL-DFO gel after one application of 10 min (**b**)

Paraloid® B72 was not detected performing either FTIR or Raman spectroscopy directly on the PHB-EL-DFO gel after one application of 10 min, also after letting the gel dry to bypass the unwanted spectral information related to the solvents present in the system (i.e., ethyl lactate and water). Therefore, the gels were submitted to extraction by acetone to solubilise deposits of the acrylic coating potentially present on the used systems. The collected extraction residue could be ascribed to Paraloid® B72 through both FTIR (data not shown) and Raman spectroscopy, when compared to the reference spectrum of the material, as reported in Fig. 7. These data confirmed that the PHB-EL-DFO formulation could tackle the organic coating already with a short-time application. Furthermore, Paraloid® B72 was successfully absorbed by the gel during the cleaning process.

Raman spectroscopy was performed on each treated area of the mock-up replicates, both before and after gel application, to monitor the cleaning performance of the PHB-EL-DFO gel.

The analysis detected Raman shifts typical of iron oxyhydroxides such as lepidocrocite γ-FeOOH (peaks at 248, 375, 526, and 650 cm^−1^) and goethite α-FeOOH (245, 309, and 387 cm^−1^) on the surface of the untreated mock-ups (SM-Figure 2) [65]. Sporadically, ferric chloride hexahydrate, utilised during the chemical ageing procedure, could be recognised by the signals at 145, 236, 254, 295, and 413 cm^−1^, comparing to the reference spectrum (SM-Figure 2). Additional bands around 598, 836, 863, 1023, 1097, and 1111 cm^−1^ could be ascribed to Paraloid® B72, when compared to the reference spectrum collected (SM-Figure 2). No further diagnostic Raman shifts could be discerned due to fluorescence interference in the spectral region at higher wavenumbers.

After gel application, the collected Raman spectra did not show the presence of Paraloid® B72 on the metal surface, potentially due to either a response under the Raman limit of detection or a complete removal of the acrylic coating. On the contrary, both lattice vibrations, related to bare steel, and remaining iron oxyhydroxides could be detected (SM-Figure 3). It is not straightforward to quantify the presence of iron corrosion compounds to compare before and after cleaning. Indeed, even though the intensity of a Raman peak is proportional to the analyte concentration, factors such as matrix absorption, surface inhomogeneity and laser beam focusing make the quantitative interpretation of Raman data complicated [66].

Overall, no wavenumber shifts or new Raman bands were observed on the treated mock-ups, revealing no modification in the corrosion phases that could potentially derive from the interaction with the PHB-EL-DFO gel. In particular, in the spectral range where Fe-DFO complexes have the strongest Raman peaks (i.e., 500–1600 cm^−1^), no evident diagnostic vibrational bands were detected, thus underlining the absence of eventual cleaning residues.

**Fig. 8:**
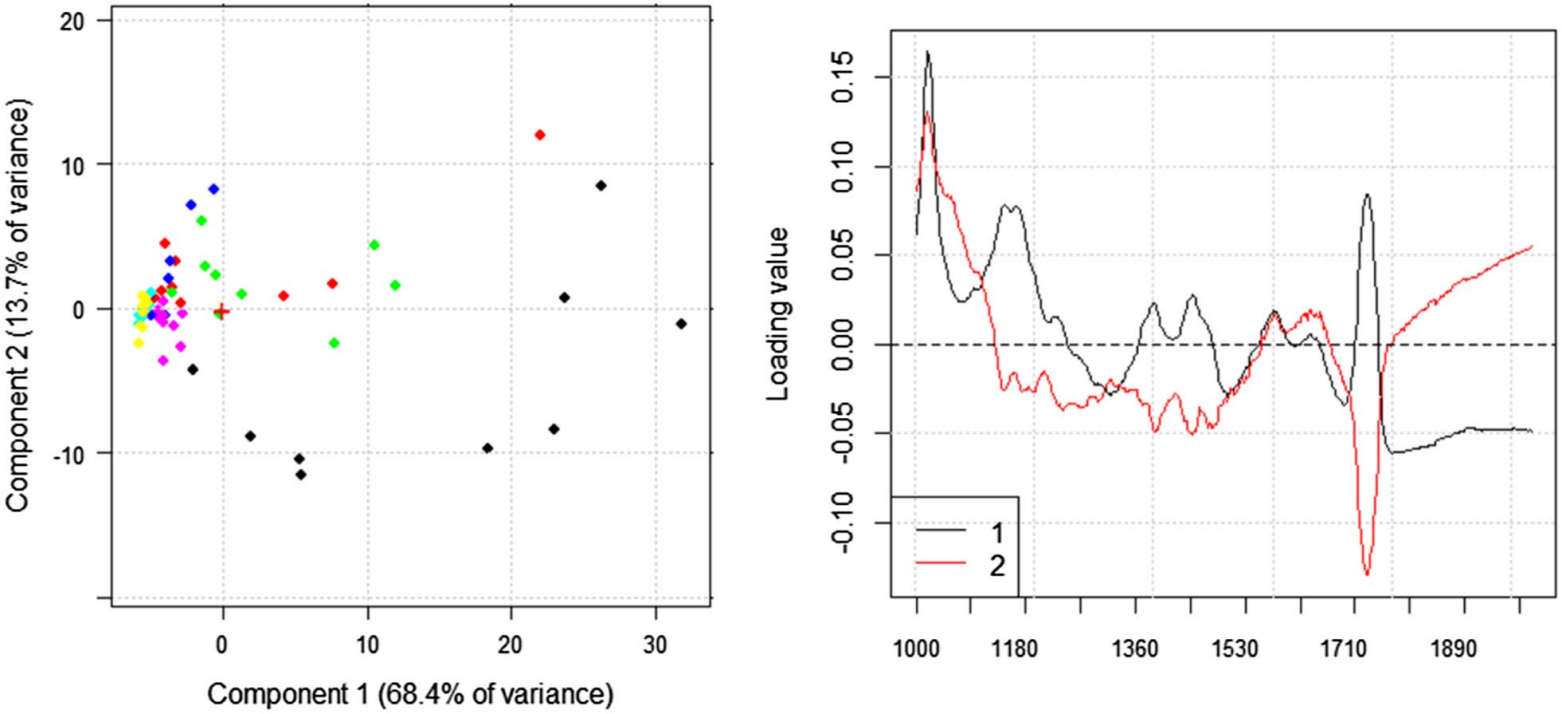
Score (left) and loading (right) plots obtained from PCA applied to FTIR spectra recorded on chemically aged mild steel mock-up, coated with Paraloid® B72, cleaned by one 10-min (black), two 10-min (red), one 20-min (green), two 20-min (blue), one 30-min (purple), and two 30-min (turquoise) gel applications, and untreated bare mild steel coupons (yellow)

Finally, FTIR spectroscopy in reflectance mode was applied on mock-up replicates to monitor the effect of different PHB-EL-DFO gel application times. The resulting data were processed using Principal Component Analysis (PCA) to facilitate the interpretation of the collected information and identify differences related to the various application times and reiterations. Specifically, multivariate analysis was applied to FTIR spectra as the technique could provide identifiable spectral features for both steel corrosion (i.e., lepidocrocite) and organic coating (i.e., Paraloid® B72) present on the model samples, making it effective for monitoring the double-cleaning action of the gel. In Fig. 8, the PC1 versus PC2 scatter plot provides information on the effect of the different cleaning times and reiterations. FTIR data from bare mild steel coupons are included as references in the plot. The related loadings plot for PC1 vs PC2 displays the spectral variables (i.e., wavenumbers) more involved in the discrimination (i.e., clusters position along the PC axes). Specifically, variables between 1710 and 1740 cm^−1^ are attributed to the absorption band of Paraloid® B72 at about 1727 cm^−1^ which is related to C=O stretching vibration [67]; variables between 1000 and 1040 cm^−1^ may be related to iron corrosion products, which were specifically tracked by the Fe-OH bending vibration at 1025 cm^−1^, characteristic for lepidocrocite [68]. Consequently, the residual presence of lepidocrocite and Paraloid® B72 after cleaning could be displayed along PC2 positive and negative values, respectively (Fig. 8). This outcome allows a clear differentiation of the mock-up areas cleaned by one 10- and 20-min (Fig. 8, black and green, respectively) and two 10-min (Fig. 8, red) gel applications from the others and, most importantly, from the cluster of bare mild steel coupons used as controls (Fig. 8, yellow). In addition, the result showed an overlapping of FTIR data related to bare mild steel coupons, used as reference, and mock-up areas treated with the PHB-EL-DFO gel for 30 min with a reiteration, indicating this cleaning protocol as the best performing on the mock-ups used in this study. This evidence suggests a successful cleaning level achieved by the innovative system with effective removal of Paraloid® B72 and iron corrosion products, thanks to the concurrent action of ethyl lactate and DFO englobed in the gelled formulation.

Finally, FTIR mapping in reflectance mode was performed on mock-up replicates after cleaning to check the presence of potential treatment residues. In particular, the FTIR band at 1045 cm^−1^ was considered as diagnostic for both DFO and Fe-DFO complexes (i.e., C–O stretching and resonative O=C–N bond) and distinguishable from other vibrational bands in the considered spectral range [64]. The resulting FTIR chemigrams did not indicate the presence of DFO-related post-cleaning residues on the treated areas of the mock-ups (SM-Figure 4).

## Conclusions

The green organogel proposed in this study introduces an innovative versatile way of cleaning altered indoor iron-based artefacts. The PHB-EL-DFO formulation was designed using exclusively biologically derived, biodegradable, and low or non-toxic components, in respect with several Green Chemistry principles [37]. Additionally, a new protocol for the simultaneous cleaning action on organic substances (i.e., acrylic coating) and inorganic compounds (i.e., iron corrosion) is explored. The research was aimed to optimise working time and reduce the amount of materials usually employed by metal conservators for the individual treatment of similar cases, while proposing a greener attitude in their daily practice. The study demonstrated that the PHB-EL-DFO gel formulation allows good spatial definition and an easy modulation of the cleaning, varying application time and, especially, gel reiteration according to specific object conditions and conservation purposes. A multi-modal approach was implemented to characterise the newly developed organogel and assess its efficiency and reliability on model samples containing both iron corrosion products and an acrylic resin (i.e., Paraloid® B72) to be removed. Micro-FTIR spectroscopy, supported by Principal Component Analysis (PCA), proved to be the most appropriate non-invasive technique for monitoring the cleaning action on the mild steel mock-ups.

The design of a renewable and non-harmful organogel for the cleaning of historical iron heritage aims to promote research and the implementation of greener and more sustainable practices in metal conservation. In particular, the collaboration with CRs for the evaluation and comparison of the cleaning performance of the PHB-EL-DFO gel to traditional methods and active agents (e.g., mechanical abrasion, EDTA) is envisioned to verify the suitability of the proposed solution in metal care interventions.

### Supplementary Information

Supplementary Material 1.

## Data Availability

The datasets generated and/or analysed during the current study will be available, once published, in the OLOS portal/Organizational unit: HE-Arc—Research Unit Arc in Conservation-Restauration/HELIX.

## Abbreviations

CRs: Conservator-restorers
PHB: Poly-3-hydroxybutyrate
EL: Ethyl lactate
DFO: Deferoxamine B
SEM: Scanning electron microscopy
TGA: Thermogravimetric analysis
XRF: X-ray fluorescence spectroscopy
FTIR: Fourier-transform infrared spectroscopy
PCA: Principal Component Analysis
