# Supplementary material for: Innovative perspective for the cleaning of historical iron heritage: novel bio-organogel for the combined removal of undesired organic coatings and corrosion
Source: Herit Sci. 2024 Jun 5;12(1):181. doi: 10.1186/s40494-024-01288-0 (PMC11150318; doi:10.1186/s40494-024-01288-0)
Supplement: Supplementary file 1 — Supplementary Material 1. [file 40494_2024_1288_MOESM1_ESM.docx]

**Supplementary materials**


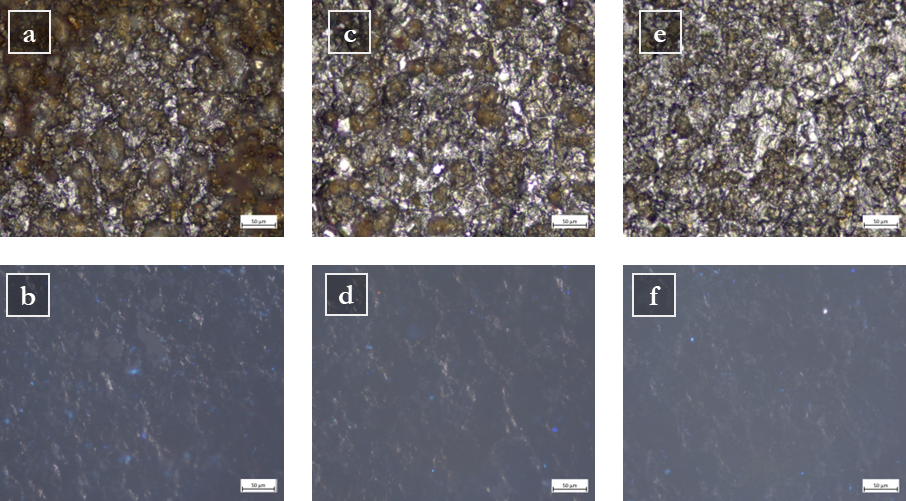


**SM-Figure 1** Optical microscope images (20 × original magnification) in bright field (top row) and under UV light (bottom row) of chemically aged mild steel mock-up, coated with Paraloid® B72, after two gel applications of 10 (a, b), 20 (c, d), and 30 (e, f) minutes, respectively. The scale bar indicates 50 µm.


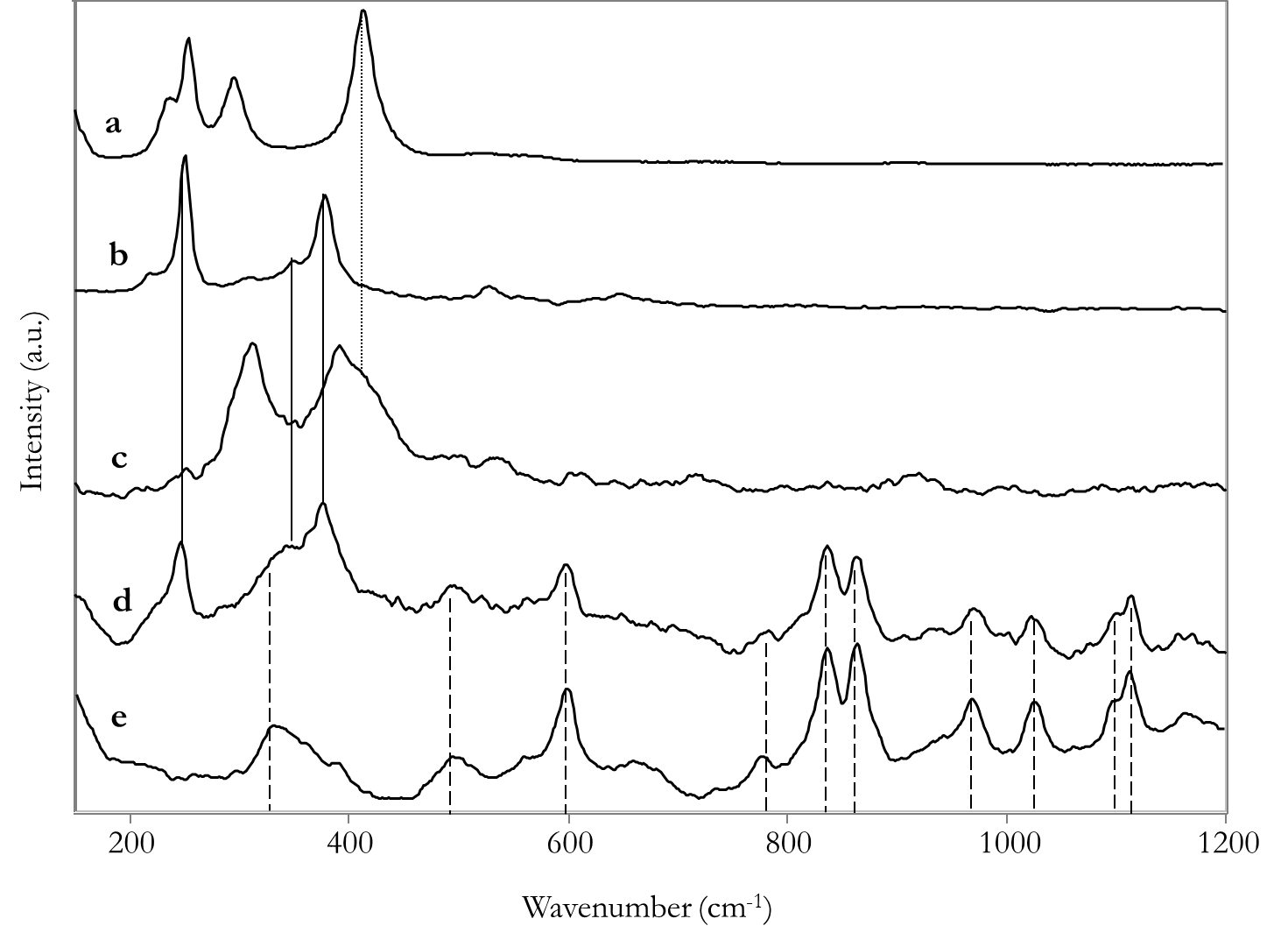


**SM-Figure 2** Raman spectra of ferric chloride hexahydrate (a), mild steel mock-up after chemical ageing (b, c), mild steel mock-up after chemical ageing and Paraloid® B72 coating (d), and Paraloid® B72 (e). Raman bands common among the spectra and related to ferric chloride hexahydrate (a), mild steel chemically aged (b), and Paraloid® B72 (e) are highlighted by dotted**,** solid, and dashed lines, respectively.


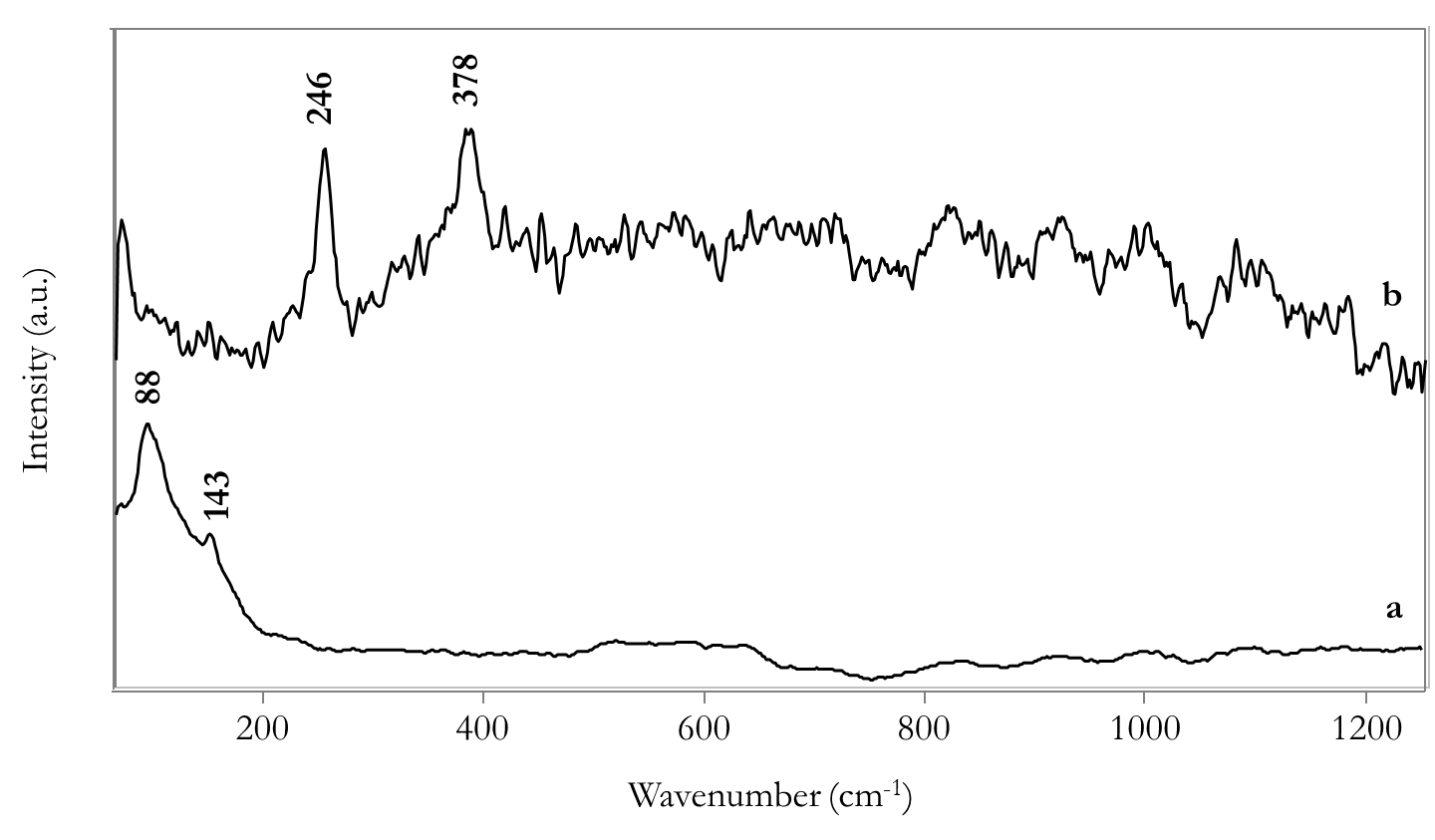


**SM-Figure 3** Raman spectra collected from chemically aged mild steel mock-ups, coated with Paraloid® B72, after cleaning. Typical signals related to lattice vibrations (i.e., bare steel) (a) and remaining iron oxyhydroxides (i.e., lepidocrocite) (b) are reported in the figure*.*

| 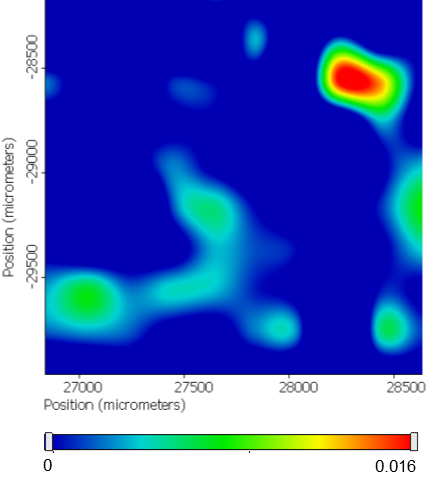 | 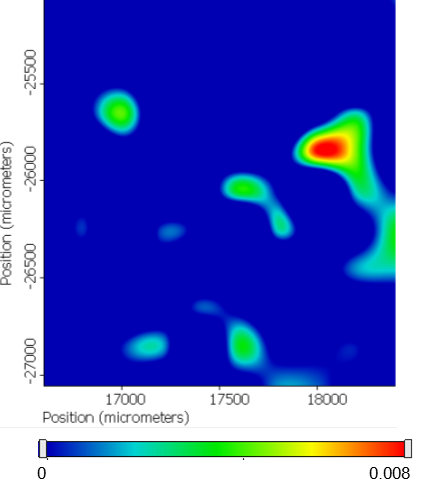 |
| --- | --- |
| (a) | (b) |
|  |  |
| 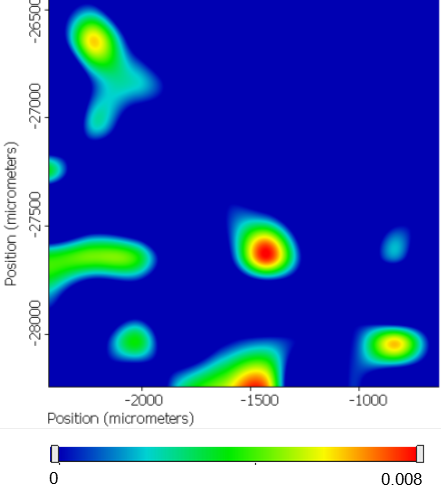 |  |
| (c) |  |

**SM-Figure 4** FTIR chemigram maps collected in reflectance mode on the surface of mock-ups after 10- (a), 20- (b), and 30-minute (c) cleaning protocols. Resulting maps when considering the FTIR signal at 1045 cm^-1^ related to both DFO and iron-DFO complexes. Absorbance scale bar reports values from min (blue) to max (red).
